# Surgery with Limited Resources in Natural Disasters: What Is the Minimum Standard of Care?

**DOI:** 10.1007/s40719-018-0124-4

**Published:** 2018-03-19

**Authors:** Miguel Trelles Centurion, Rosa Crestani, Lynette Dominguez, An Caluwaerts, Guido Benedetti

**Affiliations:** 1 Surgical Care Unit, Médecins Sans Frontières, Rue de l’Arbre Bénit 46, 1050 Brussels, Belgium; 2 Emergency Medical Unit, Médecins Sans Frontières, Rue de l’Arbre Bénit 46, 1050 Brussels, Belgium; 3 Health Structure Unit, Médecins Sans Frontières, Rue de l’Arbre Bénit 46, 1050 Brussels, Belgium; 4 Operational Research Unit, Médecins Sans Frontières, 68, Rue de Gasperich, L-1617 Luxembourg, Luxembourg

**Keywords:** Médecins sans Frontières, Doctors Without Borders, Surgery, Anesthesia, Natural disasters, Limited resources, Minimum standards, surgical care quality

## Abstract

**Purpose of Review:**

In a challenging scenario, such as in the aftermath of a natural disaster, minimum standards of care must be in place from the moment surgical care activities are launched.

**Recent Findings:**

Natural disasters cause destruction and human suffering, especially in low- and middle-income countries, which suffer the most when exposed to their consequences. Health systems can quickly get overwhelmed and can collapse under the burden of injured patients during this event, while qualified surgical care remains crucial. Medécins Sans Frontières (MSF) has a vast experience providing surgical care after natural disasters, and quality is assured through the Donabedian model. Minimum structure standards are put in place from the beginning of an emergency response, together with standard operating procedures providing guidance to professionals working in challenging conditions.

**Summary:**

MSF believes that it is always possible to deliver surgical care, ensuring the best possible quality guaranteeing adequate levels of structure and process. The “do no harm” principle must always be respected as adherence to medical ethics is a must in any context, even a challenging one.

## Introduction

A disaster can be defined as “an unforeseen and often sudden event that causes great damage, destruction and human suffering; overwhelming local capacity and necessitating a request at the national or international level for external assistance” [1]. These events can be natural or man-made: while the first is caused by natural processes of the earth (e.g., earthquakes, hurricanes, floods), man-made disasters are caused by deliberate human action or negligence (e.g., armed conflicts, industrial and nuclear incidents, fires). All inhabited areas around the globe are prone to natural disasters but the toll in victims significantly differs due to the degree of emergency preparedness and development of a given country. Therefore, low- and middle-income countries (LMICs) are the most exposed to the negative impact of such natural events.

LMICs have limited capacities to natural disasters as they do not have enough resources to meet them [2••]. The provision of adequate health care remains critical in such scenarios as the health systems of many LMICs are not fully developed and still face scarcity and inadequacy of health infrastructures, shortage of material and resources, paucity of qualified human resources, and limited access to care due to geographical and economic barriers, among others [3–6, 7•]. Consequently, chronically fragile health systems can get quickly overwhelmed and they are likely to collapse in the aftermath of a natural disaster. Abruptly, the population needs swell, while health infrastructures are damaged, making an already insufficient offer of care even more inadequate. The immense initial needs are solely handled by the overwhelmed local health forces [8].

The burden of injuries is usually heavy as a consequence of a natural disaster, e.g., in a high-magnitude earthquake. Therefore, the availability of prompt and qualified surgical care is crucial, though the provision of surgical care in the world is not equal. Most of the unmet needs for emergency and essential surgical care are concentrated in LMICs: the poorest third of the world population only benefits of around 4% of all the performed surgical procedures [9, 10•, 11, 12]. That is the reason why it is expected that the provision of surgical care after a natural disaster is much more critical than in normal circumstances, with the usual burden of surgical/obstetric conditions that are also in need of urgent care are still present in addition to the new needs as a result of the natural disaster [13]. Consequently, international support is substantially needed.

Médecins Sans Frontières (MSF), known in English as Doctors Without Borders, is an international non-governmental, non-profit, and humanitarian medical organization, which provides health care to vulnerable populations, including the ones affected by natural disasters for 45 years. The organization intervenes whenever populations are in need of help providing qualified assistance regardless of race, religion, or political convictions. Their actions are driven by medical ethics maintaining independence, neutrality, and impartiality. MSF has a vast experience in providing surgical care in the aftermath of natural disasters. MSF offers care free-of-charge, following standard operating procedures, and adapting strategies and approaches to any given context.

## Health-care Quality

The World Health Organization (WHO) defines quality of care as “the extent to which health care services provided to individuals and patient populations improve desired health outcomes. In order to achieve this, health care must be safe, effective, timely, efficient, equitable and people-centred” [14]. It is important to recognize that in any circumstance, including the aftermath of a natural disaster and under precarious conditions, health care should always be given following standards of quality; otherwise, it cannot be compatible with medical ethics [15].

Measuring the quality of provided health care has been debated for several decades since the concepts of quality assurance and medical liability became more important. Presently, the Donabedian model is recognized as the most common framework in measuring the quality of health care [16]. It is a conceptual model where three categories are evaluated: structure, process, and outcomes. The structure category considers the context where health care is given: infrastructure, human and material resources, and medical equipment. Process considers the “how” health care is provided. Finally, outcomes describe the results of health care, and it is directly linked to the first two categories: structure and process [17]. The Donabedian model was initially applied to evaluate medical care, and presently, it is also widely used in appraising the provision of surgical care [18, 19••]. Quality in the provision of surgical care needs to consider these three proposed dimensions. Unfortunately, measuring the outcomes of the provided surgical care during natural disasters is difficult, as patients might move to another place, or more importantly, the health providers might soon leave the scene [20•]. Surgical care in the aftermath of natural disasters in LMICs is usually provided by a number of international medical teams working for a limited period and sometimes in temporary health structures. Big efforts should be done to provide good standards in structure and process, putting the basis for good outcomes, which remain difficult to measure [21].

## Standards of Surgical Care

Defining the minimum standards of care that should be followed when dealing with the health of an individual is challenging. Indeed, a set of minimum standards is essential to assure the safety of patients and the quality of provided care. In a challenging scenario, such as in the aftermath of a natural disaster, minimum standards of care must be in place from the moment surgical care activities are launched. The medical principle of “do no harm” must also be respected.

During the last century, several attempts were done to standardize surgical care, mainly by influential big health-care institutions in high-income countries. In 2003, the WHO identified criteria for structure and processes relevant to the provision of surgical care [22]. This was an important step towards the standardization of surgical care at district hospital level in LMICs. Afterwards, guidelines about infrastructure and supply for different levels of health care and about surgical care capabilities were proposed for surgery and anesthesia [23]. Nonetheless, these minimum standards did not comprehensively address several aspects related to structure and process. Furthermore, they were intended to be used in routine surgical care and not in complex situations as during the occurrence of a natural disaster.

The need of minimum standards to provide quality surgical care arose again after the devastating January 2010 earthquake in Haiti, where the number of deaths and injured was extremely high and damages to health-care infrastructures were foremost. As the earthquake hit the capital city, the health-care system became immediately overwhelmed and several international medical teams arrived to provide assistance. The support offered by these teams was vital to the local population but also brought several questions about their impact. Some authors later identified problems in (i) the adherence to international guidelines based on the analysis of monitoring and surveillance data, (ii) the organization and governance of provided services, (iii) difficulties of the international staff to adjust to harsh conditions, (iv) balancing the available capacity of response with the high population’s demand for care while maintaining acceptable standards, (v) the poor coordination among health providers, and (vi) the unavailability of ready-to-use emergency kits [24–27]. Importantly, it was also experienced how international medical teams sometimes could not cope with certain health problems due to the existing gap between their training and the health conditions faced in such a challenging context [28].

As an answer to the existing vacuum of clear operational indications, the WHO commissioned a guide of minimum standards for international teams to be deployed after a disaster. Such a tool was eventually published in 2013 [29]. This document does not only provide an in-detail list of standards, but importantly, classifies the provided medical services as an (i) outpatient emergency care, (ii) inpatient surgical emergency care, and (iii) inpatient referral care, following their capacity to provide surgical care. When typhoon Haiyan hit the Philippines on November 2013, the local government in coordination with the WHO tried to monitor the deployment of international relief agencies following the new published document. When evaluating the performance of these agencies during this disaster, the guide was efficient in classifying the teams at arrival and prioritizing assistance to the affected areas. Unfortunately, it was not possible to assess whether the necessary minimum standards of health care were in place [30]. This might have been a problem of timing as the new proposed tool was just published and further utilization of it was needed under real conditions.

## Médecins Sans Frontières Experience and Standards of Surgical Care

During the last years, MSF has been a key humanitarian actor in natural disasters, especially when the needs in secondary level are important. In these situations, MSF offers assistance to the direct victims of a disaster (e.g., orthopedic care) and also to the indirect victims that are suffering from the lack of access to surgical care (e.g., obstetric care, surgical acute conditions). Life- and limb-saving surgical care is the first priority when launching activities. Only when the best possible standards are in place is essential and specialized surgery (including complex orthopedic care) offered [31]. Such an upgrading of the offer of care is possible when the context already allows handling an increasing higher caseload without compromising life-saving procedures and when surgical care is already well functioning. Whenever surgical care is no longer offered after the emergency phase, MSF makes efforts to assure an appropriate follow-up of the treated patients. These principles are also applicable and should be instituted when providing health care during man-made disasters.

The prompt provision of adequate human and material resources is an important challenge to MSF and other international organizations, as natural disasters are not foreseeable [32]. Qualified surgeons and anesthesiologists are deployed according to the needs in a given context, as well as scrub and ward nurses. Anesthesiologists might face difficulties using drugs and basic biomedical devices that are different from their routine practice, but the required skills remain the same always [33]. On the contrary, surgeons trained and working in high-income countries might sometimes lack the required surgical skills (e.g., such as how to perform a cesarean section) as surgical training in developed countries is getting more and more specialized [34, 35]. Fortunately, today, this problem is being tackled by different organizations and universities worldwide, including MSF, where dedicated trainings are offered to upgrade the skills of surgeons willing to help in humanitarian relief operations [36, 37]. Concerning material resources and their supply, MSF has different warehouses around the world where items are pre-emptively stored, packed, and ready to be delivered following a standardized approach. MSF central distribution centers are located in Bordeaux, France, and Brussels, Belgium, while emergency preparedness stocks are stored in the countries where MSF has running projects. It should be noted that considerable care and related resources are required in dealing and managing injured patients [38].

Following the Donabedian quality model for health care, MSF commits to ensuring that surgical care is performed in an adequate environment or structure. These include an operating room protected from external agents, enough water resources of reliable quality, electricity, adequate circuits for waste disposal including the management of dead bodies, strict infection prevention and control measures, and sterilization capacity. Moreover, a basic set of biomedical devices is provided since commencement of surgical activities including operating room lights, oxygen concentrators, pulse oximeters, and suction devices. Whenever possible, electrosurgical units and multiparameter monitors are also deployed. Essential drugs and consumables are available following a strict standardized procurement and following protocols. As injuries are a direct consequence of natural disasters, blood transfusion capacity is also ensured.

At the time activities are launched, MSF obliges to apply standard operating procedures for most of the performed activities. This kind of approach improves the quality of care as it provides guidance to people working in challenging conditions, and each having different knowledge and experience. Standard operating procedures are safe, simple, and effective and adapted to operational constraints. Appropriate and standardized data management processes are put in place, including surgical surveillance, line-lists for monitoring, and patient files. After a patient undergoes a surgical/orthopedic treatment, post-operative care is given, including early physical and functional rehabilitation, as it improves patients’ outcomes [39, 40••]. Privacy of the patients is respected as much as possible. Written informed consent for a surgical procedure is obtained from the patient, after explaining the nature of the treatment, and if not possible, from a family member. Whenever contextual difficulties or the life-threatening condition of the patient do not allow obtaining a consent, is documented and justified.

In order to ease the deployment of surgical care after a natural disaster, MSF has developed three packages during the last years: (i) a very basic one called Rapid Intervention Surgical Kit (RISK), (ii) a more complex Rapid Deployable Surgical Unit (RDSU), and (iii) a Modular Field Hospital (MFH) including inflatable tents or semi-permanent prefabricated modules [41]. These packages are flexible enough to be adapted according to specific contextual needs and are used to provide care for both trauma and non-trauma patients, including obstetric care. The approach is phased and the different features of each solution are given in Table 1:

- The Rapid Intervention Surgical Kit (RISK) is basically an isolated operating room and can be fully operational in hours with autonomy for 3 days (5 major surgeries per day). Pre-sterilized surgical sets are available in order to perform surgery almost immediately while sterilization is set up. It is easy to transport as it fits in a vehicle (e.g., a four-wheel off-road vehicle) or helicopter. A surgeon, an anesthesiologist, and a scrub nurse are deployed with it. An emergency medicine physician and a nurse are also part of the team to provide pre- and post-operative care.
- The Rapid Deployable Surgical Unit (RDSU) can be considered as an isolated surgical department that includes emergency medicine services. It can be fully operational within a week. It is easily set up and maintained as a field hospital, and it can stand alone or be adjacent to an existing health facility (e.g., a local health center). As it is made up of inflatable tents, it follows a modular concept giving flexibility according to needs. Moreover, there is the possibility to include an X-ray capacity.
- The Modular Field Hospital (MFH) is a complete hospital including other services aside from the surgical and emergency medicine services. It is fully operational in a month. Using inflatable tents versus other solutions is preferred due to transport constraints. MSF is presently testing the use of surgical operating theaters in containers on trucks. Excellent examples of the experience of its use are after the Haiti earthquake in January 2010 when MSF deployed a 100-bed hospital in Port-au-Prince and after the Philippines was hit by typhoon Haiyan in November 2013 when MSF deployed a 45-bed hospital in Tacloban.

**Table 1:** MSF solutions for deployment of surgical care after a natural disaster

	Rapid Intervention Surgical Kit (RISK)	Rapid Deployable Surgical Unit (RDSU)	Field Modular Hospital (FMH)
Concept	- It is used in the early phase of response when there is a need to have a fast response and when accessibility is difficult.	- It is used in the early phase of response when it is needed to increase the surgical capacity or to add capacity to a local health structure.	- It is a 3rd level health-care facility used as a general hospital or to provide specialized surgical care.
Timelines	- It can be deployed in 1 day from the occurrence of the disaster. - It can be used for 3 days without refilling or re-stocking	- It can be deployed in days from the occurrence of the disaster. - It can be used for 30 days or more, according to the context.	- It can be deployed in 15 days from the occurrence of the disaster, if the conditions are optimal. - It can be used for several months, according to the context.
Features	- The main module has basic supplies and 1 tent. It has a weight of 570 kg and a shipping volume of 3.5 m^3^. - Additional modules are used for specific needs. They have a weight of 175 kg and a shipping volume of 1.5 m^3^. - The tent serves as the operating room, is around 15 m^3^ and it aims to provide a secure place where to perform surgery. It can be placed inside a room that is not prepared for surgical activities.	- The main module of 5 tents has dedicated areas for pre- and post-operative care, operating room and sterilization. It has a weight of 30 tons and a shipping volume of 160 m^3^. - The additional modules consider the possibility of adding a second operating room, X-rays, and areas for triage and dressings. They have a weight of 5 tons and a shipping volume of 25 m^3^. - The tents measure 7.5 m × 5.6 m.	- The standard hospital of 9 tents has dedicated areas for triage and emergency care, including observation. Also, areas for pre- and post-operative care are available, including intensive care. There is an operating complex that can host 2 operating rooms and an area for sterilization. It is possible to have up to 3 wards with 16 beds per tent. - The modular concept gives great flexibility for deployment. Therefore, it is possible to have a reduced or an enlarged version of the standard solution. - The tents measure 12.0 m × 8.0 m.
Activities capacity	- Resuscitation and pre-operative care - Damage control surgery - Trauma surgery - Visceral and obstetrical emergencies - Basic orthopedic care including external fixation - Post-operative care - Delayed primary closure of wounds - Basic laboratory: hemoglobin, glucose, urine test strips - Ultrasound	It offers the same activities as the RISK and also: - Triage and medical emergencies - Emergency deliveries and post-partum complications - Wound care - Head trauma - Palliative care - Laboratory: blood count, biochemistry, microscopy, etc. - X-rays	It offers the same activities as the RDSU and depending on the context and needs also - All kind of surgical and medical emergencies, including observation - Essential surgery - Specialized surgery including complex orthopedic care - Intensive care without assisted ventilation - Physical therapy - Mental health - Laboratory: antibiogram - Outpatient consultations
Needed minimum staff	5–6 people: surgeon, anesthetist, scrub nurse, emergency medicine physician, polyvalent nurse, logistician and a coordinator.	16–26 people: 4 surgeons, 4 anesthetists, 4 scrub nurses, 2 emergency medicine physicians, 1 medical general doctor, 1 head nurse, 4 polyvalent nurses, technicians for the laboratory and X-rays, several logisticians, administrator and coordinator.	This temporary solution works as a general hospital and therefore, the staff needed is the same at that of a normal setting.

These three types of surgical response are temporary and aim to begin activities as quick as possible where there is lack of infrastructure and supply. Ancillary services (e.g., sterilization, laboratory) are also foreseen from the beginning. Importantly, when providing surgical care in a place affected by a natural disaster, it must also be considered that the relief teams should be autonomous in terms of transport and supply (including water) to avoid competing with the local population for basic means and needs.

Alongside the surgical team, there are other paramedical and non-medical professionals working in parallel to deliver quality surgical care to the patients. General practitioners, nurses and midwives, psychologists and physiotherapists, and other paramedical professionals are just as important and ensure a holistic approach to the injured patient. Non-medical professionals as architects and engineers, water and sanitation specialists, electricians, constructors, and other logisticians are essential to set up an emergency response and ensuring the best possible standards of quality. They are sometimes deployed even before the arrival of the surgical team. Human resources and financial administrators are also key players. All are indispensable and work as a team in humanitarian medical relief work. During an emergency intervention, the ratio of local and international staff is around 15 to 1. In the event of a natural disaster, local health-care teams are the first responders and the ones facing the greatest difficulties. Despite being victims themselves of the natural disaster, they are the first to provide assistance to other affected victims.

## Conclusions

A list of required minimum standards to perform surgical care during natural disasters is already available since 2013 when the WHO developed a guide for international medical teams. As a humanitarian organization, MSF has been providing surgical care to populations in need for 45 years and it follows the Donabedian model of quality assurance. While ensuring proper structure and process standards, life-saving and limb-saving surgical care remains the first priority after a natural disaster. The offer of essential and specialized surgery at a later stage, when a higher caseload can be managed, should not compromise the quality of care. Early rehabilitation of patients and a proper follow-up of operated cases should be established also.

The structure component of quality care according to the Donabedian model includes adequate human resources, appropriate infrastructure with water and electricity supply, biomedical devices, and a guaranteed supply of validated drugs and consumables. Unfortunately, there is often a gap between the needs in humanitarian surgery and the surgical training programs in high-income countries, limiting the availability of polyvalent surgeons for such type of events. As such, there is space for the development of ad hoc trainings. At the same time, the procurement on a short notice of drugs and consumables is a difficult task, and humanitarian organizations might consider having ready-to-use stocks prepositioned in different areas of the world. As natural disasters might damage the local health infrastructure, possibilities to have easy-to-use deployable operating rooms remain a relevant option. Sterilization is a key component of surgical care and its adequate function should be timely ensured. Also, international medical teams should be autonomous in order not to burden local authorities or to compete with the local population for certain resources, such as water and transport.

The process component of quality care according to the Donabedian model is assured by adherence to standard operating procedures that are very important in contexts where international personnel might be from different backgrounds. Correct recordkeeping and data collection are recommended to be in place from the beginning. Obtaining a written informed consent before surgery from the patients or their relatives remains of capital importance.

Any international medical team arriving in the aftermath of a natural disaster needs to acknowledge that local teams were the first responders, while also being victims. Collaboration with them is a must.

MSF considers that during the aftermath of a natural disaster, it is always possible to deliver surgical care to the population in need, while ensuring the best possible quality. This includes adapting to the specific context, guaranteeing proper structure and process components, and mitigating the obvious difficulties in measuring outcomes in such challenging scenario. The “do no harm” principle must be respected at all times as adherence to medical ethics is paramount in any context, even a challenging one. This value drives the provision of surgical care within MSF.
